# Reproduction of human blood pressure waveform using physiology-based cardiovascular simulator

**DOI:** 10.1038/s41598-023-35055-1

**Published:** 2023-05-15

**Authors:** Jae-Hak Jeong, Bomi Lee, Junki Hong, Tae-Heon Yang, Yong-Hwa Park

**Affiliations:** 1 Department of Mechanical Engineering, Korea Advanced Institute of Science and Technology, Daejeon, 34141 Republic of Korea; 2 Department of Electronic Engineering, Korea National University of Transportation, Chungju-si, Republic of Korea

**Keywords:** Blood flow, Biomedical engineering

## Abstract

This study presents a cardiovascular simulator that mimics the human cardiovascular system's physiological structure and properties to reproduce the human blood pressure waveform. Systolic, diastolic blood pressures, and its waveform are key indicators of cardiovascular health. The blood pressure waveform is closely related to the pulse wave velocity and the overlap of the forward and reflected pressure waves. The presented cardiovascular simulator includes an artificial aorta made of biomimetic silicone. The artificial aorta has the same shape and stiffness as the human standard and is encased with a compliance chamber. The compliance chamber prevents distortion of the blood pressure waveform from strain-softening by applying extravascular pressure. The blood pressure waveform reproduced by the simulator has a pressure range of 80–120 mmHg, a pulse wave velocity of 6.58 m/s, and an augmentation index of 13.3%. These values are in the middle of the human standard range, and the reproduced blood pressure waveform is similar to that of humans. The errors from the human standard values are less than 1 mmHg for blood pressure, 0.05 m/s for pulse wave velocity, and 3% for augmentation index. The changes in blood pressure waveform according to cardiovascular parameters, including heart rate, stroke volume, and peripheral resistance, were evaluated. The same pressure ranges and trends as in humans were observed for systolic and diastolic blood pressures according to cardiovascular parameters.

## Introduction

To reproduce the blood pressure (BP) waveform and investigate the mechanism of the BP waveform generation, we developed a dedicated hardware simulator that replicates the physiological structure and physical properties of the human cardiovascular system. Before the pandemic distorted the global causes of death, according to WHO statistics, 17.9 million people worldwide died from cardiovascular diseases in 2019, accounting for 32% of all deaths in a year^1^**.** Therefore, the importance of cardiovascular health monitoring is increasing. Today, health sensors are gradually being distributed in the form of wearable devices such as smartwatches, and pulse measurements using photo-plethysmography (PPG) or electrocardiogram (ECG) to monitor cardiovascular health conditions^2–4^**.** Cardio-vascular pulse waves are monitored for various healthcare purposes, such as exercise monitoring and arrhythmia diagnosis. Systolic blood pressure (SBP) and diastolic blood pressure (DBP) mean the maximum and minimum BPs, respectively, the most important and frequently monitored health indicators for cardiovascular health management. However, BP is correlated with various cardiovascular parameters such as the stiffness of the vessel wall, pulse wave velocity (PWV), vessel diameter, and cardiovascular diseases^5^**.** Therefore, to study the causes and effects of various cardiovascular diseases systematically, studies on the BP waveform have been conducted^6–8^. It is known that the number of hypertensive patients increases with age, and one of the causes is increasing vascular stiffness with age. In both men and women, the PWV increases proportional to their ages, since the backward reflected pulse wave from the aortic bifurcation (AB), where the abdominal aorta diverges into the left and right iliac arteries, arrives earlier. As a result, the proportion of the backward reflected wave added to the forward propagating wave increases to raise the SBP, leading to hypertension and degenerative cardiovascular disease^6,7,9–11^. From clinical statistics, exercise load, heart rate (HR), stroke volume (SV), and muscle stiffness were suggested as the causes of changing BP^12^. How HR and SV change the mean arterial pressure (MAP) and pulse pressure (PP, amplitude of BP, i.e., difference between SBP and DBP) was studied through pacemaker and drug dosing^13,14^. However, in clinical studies, the control of cardiovascular parameters and corresponding data collection are almost impossible due to mostly the difficulty of invasive measurements and the bias of sample collections.

Therefore, previous studies have been conducted using various cardiovascular simulators that mimic human body physiology to study the relationship between cardiovascular system and BP waveform^15–24^. For this purpose, a series of cardiovascular simulators were developed, such as the phantom of the left ventricle-valve-aorta^15,16,25–28^, the pneumatic-based brachial pulse wave generator for calibration of a BP monitor^18,22,29,30^, the cardiovascular surgery model^31^, or the pulse wave generator for durability testing of artificial valves and stents were presented^18,32–36^. In these previous studies, only the structural shape and basic layout of the human cardiovascular system were mimicked. To reproduce the human BP waveform determined by the PWV and wave propagating and reflection mechanism, the structure of the blood vessel wall and its physical properties must be implemented close to human. To reproduce the physical properties of actual blood vessels, studies on the fabrication and characteristics of artificial blood vessels were conducted^37–40^. Rather than the entire blood vessel, only physical properties were implemented by stacking silicone layers of various stiffness in the form of sheets, or only PWV reproduction in a short straight section was studied^38,39,41^. These existing cardiovascular simulators and artificial blood vessel studies use different geometries and properties from those of the human cardiovascular system. Therefore, the overlap of the forward and reflected waves and PWV, i.e., the main parameters that determine the BP wave, are dissimilar to those of the human body. It only creates a periodically pulsating pressure waveform, which is different from the human BP waveform. Therefore, its use is limited to surgical training or durability evaluation of artificial organs in a pulsating environment, and it is impossible to study the generation mechanism and characteristics of BP waveforms of human in a precise way.

**Figure 1:**
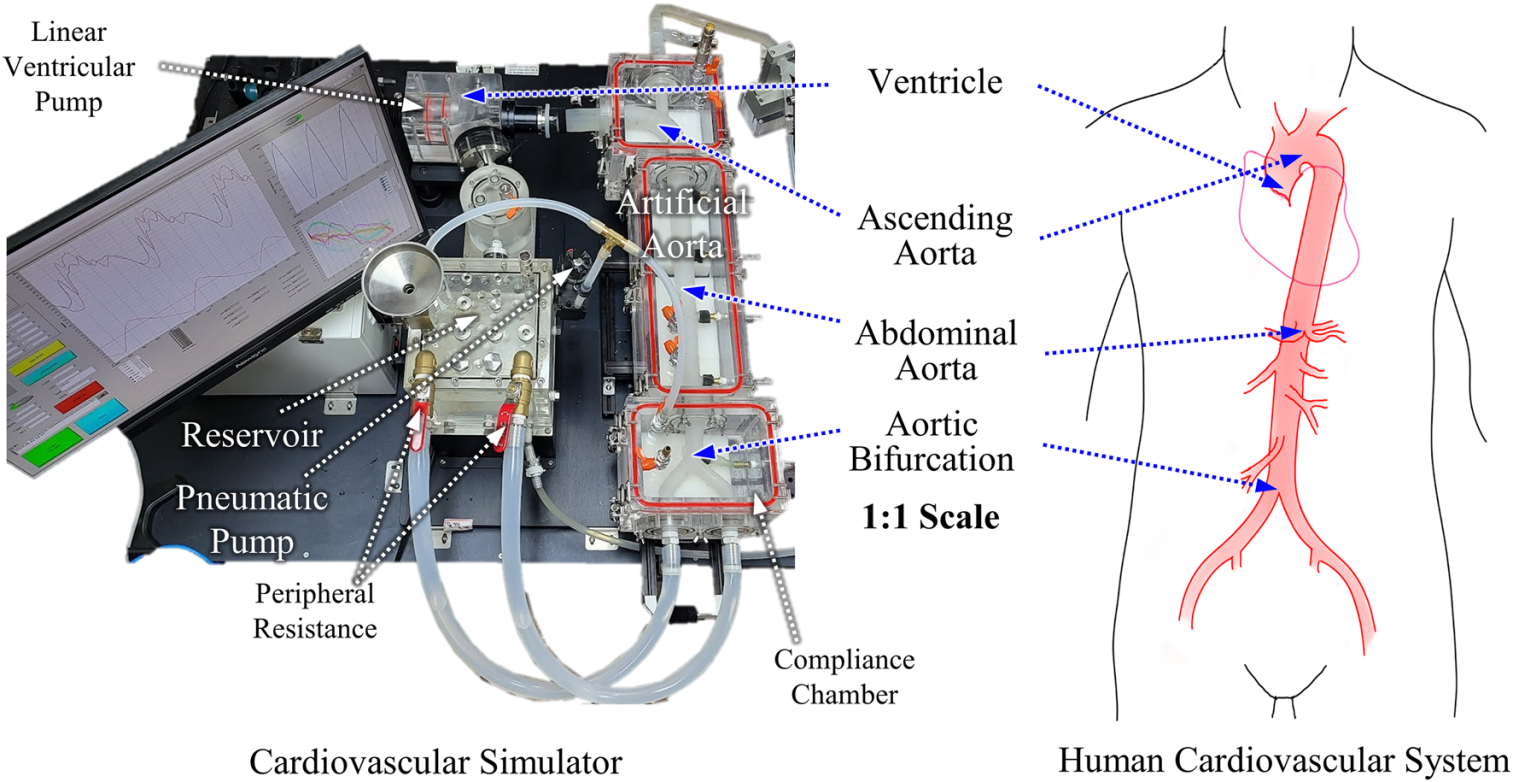
Presented cardiovascular simulator that mimics the human cardiovascular system.

Figure 1 shows the specially designed cardiovascular simulator presented in this study to overcome the limitations of the previous cardiovascular simulators. The simulator includes an artificial aorta that mimics both the structural layout and material properties of the human cardiovascular system, to produce the BP waveforms similarly as human does. The presented simulator mimics blood circulation in the cardiovascular system consisting of a linear ventricular pump that mimics the left atrium and left ventricle, aortic valve, artificial aorta, ball valves for controlling peripheral resistance (PR), and a reservoir that mimics a vein. The artificial aorta imitates the geometry of the human central aorta and is fabricated with biomimetic silicone to have the vascular stiffness of the human standard. To control the intravascular pressure (IVP) and extravascular pressure (EVP) applied to the artificial aorta, it can be pressurized from the inside and outside through the reservoir and compliance chamber, respectively, with a connected pneumatic pump. They reproduce the heart-valve-aorta relationship, PWV, and wave overlap that determine the BP waveform in human. The BP waveform generated by the simulator has a similar waveform to humans and reproduces the increase in PP and the change of the waveform as it propagates through the aorta. Changes in BP waveforms according to the changes in the cardiovascular parameters were also investigated. The increases in SBP and DBP by controlling HR and SV were compared to those of human. Changes in the BP waveform due to PR were also observed, and the trends of increase in MAP and decrease in PP were compared to those of human. Changes in the BP waveform with IVP and EVP changes were also observed. When IVP and EVP were kept the same, it was possible to change the MAP without changing the blood pressure waveform.

## Theoretical background

### Overlap of forward and reflected blood pressure waves

**Figure 2:**
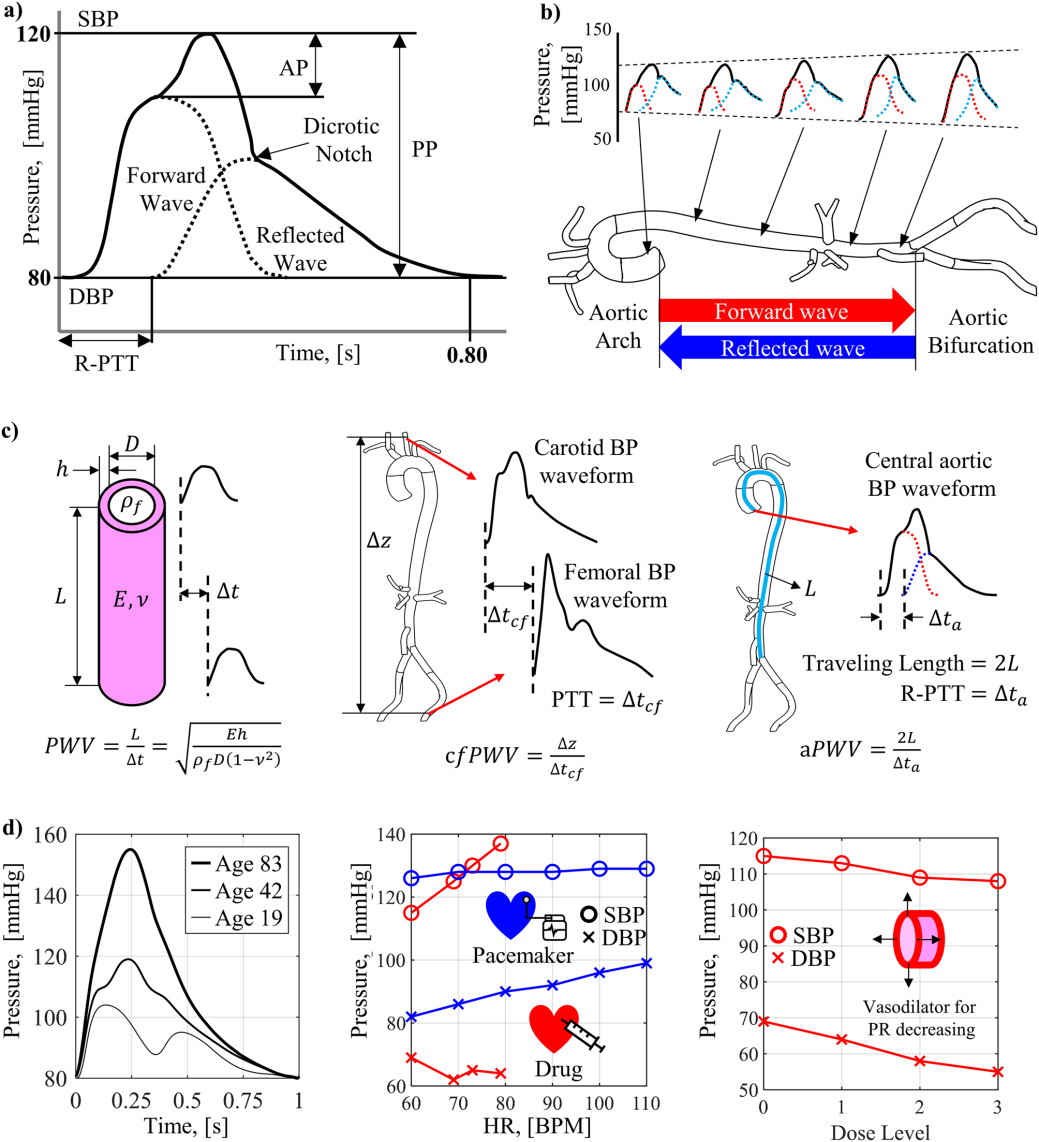
Clinical characteristics of the human BP waveform; (**a**) central aortic BP waveform formed by the overlap of forward and reflected waves, (**b**) changes in BP waves during propagation in the aorta, (**c**) definition of PWV: pressure wave propagation speed in an elastic tube, measurement of cfPWV from carotid and femoral BP wave, measurement of aPWV from central aortic BP wave, (**d**) changes in BP according to cardiovascular parameters including age, HR, and PR.

The central aortic BP waveform refers to the pressure signal measured from the aortic arch (AA), which is directed upward just after the left ventricle. Its canonical waveform and characteristics are shown in Fig. 2a^5^. The central artery BP waveform is formed by the overlap of the forward propagating wave and the backward reflected wave. Constructive interference occurs in the section where the two waves overlap. Therefore, the BP is augmented higher than the forward wave, and the increased pressure is called augmentation pressure (AP). The SBP is the highest value in the BP waveform augmented by AP. The augmentation index (AIx), the ratio of PP to AP, dependent on BP waveform, ranges from 11 to 16% in the human standard^42^. After the pressure change due to the systole ends, diastole begins, and a sudden pressure change due to the occlusion of the aortic valve appears. A slight increase in BP or an inflection point may appear due to the valve occlusion, a dicrotic notch. In overall, the BP waveform is critically affected by the reflected wave arrival time and amplitude, which determines the overlap^5^. The position where the forward wave is reflected from the aorta is the AB where the abdominal aorta divides into the bifurcated femoral artery. As shown in Fig. 2b, the closer to the AB, the shorter the time interval between the observed forward and the reflected waves, and the region where the overlap occurs becomes wider^5^. Therefore, the region where the augmentation occurs during systole is widened, the systolic peak is raised, i.e., SBP is increased.

### Pulse wave velocity (PWV)

The PWV means the propagation speed of the BP waves. The left-hand side of Fig. 2c describes the propagation speed of the pressure wave. PWV is the value obtained by dividing the distance *L* between measurement points by the pulse transit time (PTT, $\Delta t$). To understand the physical meaning of PWV, the blood vessel is modelled as a hydro-dynamic component composed of an elastic tube and incompressible fluid. The corresponding pressure wave propagation speed of the fluid in the elastic tube can be described in Eq. (1) by *Moens*^5,43^ as

1 $$PWV=\sqrt{\frac{Eh}{{\rho }_{f}D\left(1-{\nu }^{2}\right)}}$$

where the blood vessel is physically modeled with an elastic tube with elastic modulus $E$, Poisson's ratio $\nu$, length $L$, diameter $D$, thickness $h$, and the density of the incompressible internal fluid ${\rho }_{f}$. This is an equation in which the change in vessel wall thickness due to Poisson's ratio is corrected in the PWV previously proposed by *Moens*^5,7^. The elastic modulus *E* of the vessel wall, called *stiffness* afterward, is one of the key parameters related to cardiovascular health condition, which is investigated in the next section. The major propagation medium of the BP pulse is the vessel wall, and the internal blood contributes in the form of a mass loading onto the vessel wall^43^. There are studies aimed at investigating and correcting the relationship between PWV and other parameters, such as IVP and fluid viscosity^7,44–47^. The parameters that determine PWV, i.e., the stiffness *E*, and diameter *D* of blood vessels in the body, are functions of IVP. Since IVP fluctuates between SBP and DBP within a pulse, PWV also changes accordingly. Therefore, the average value of periodic pulses is commonly used^5,9,48–51^. In this study, PWV and the physical quantities used in its calculations were pulse-averaged, meaning that averaged values on time within a single pulse. However, in this study, the diameter and stiffness of the aorta were based on clinically measured values^5,9,48–51^. The artificial aorta was manufactured, and PWV was calculated based on these values, which had already considered changes caused by IVP and interactions with surrounding tissues of the in-vivo state. Moreover, the term for viscosity was not included in the calculation of PWV. Concerning the generation of the flow velocity profile, viscosity has a dominant effect in small-diameter vessels ^7,45^. But in this study, we focused on the large-diameter aorta, where the effect of viscosity is little, and inertia is dominant. This was also mentioned in *Womersley*'s study^46,47^, where the larger the diameter of the vessel, the flatter the flow velocity profile, and viscosity is negligible.

The center of Fig. 2c shows the clinically measured carotid-femoral PWV (cfPWV), which is the clinical standard for measuring human PWV^5^. The cfPWV is obtained from the BP waveforms measured from the carotid artery of the neck and the femoral artery of the thigh using a pressure cuff or tonometry probe. It is calculated by dividing the distance between two measurement points by the PTT ($\Delta {t}_{cf}$)^52–55^. According to the statistical studies of cfPWV, it is shown that the cfPWV increases with age^51^.

The right-hand side of Fig. 2c shows the aortic PWV (aPWV), estimated from the central aortic BP waveform, obtained by the forward wave from the AA and the reflected wave from the AB. The BP waveform depends on the PTT of the reflected wave (R-PTT, ${\Delta t}_{a}$), which is determined by the aPWV and distance to the AB (*L*). The aPWV is the average value of the PWV from the aortic root to the AB and the cfPWV is the average value of the PWV from the carotid artery to the femoral artery, which about 20% larger than aPWV because it includes the femoral artery, which has a small diameter and large PWV^5,9^.

The PWV is an effective indicator of 'vascular age' because the aging of blood vessels induces change in stiffness, which is directly related to PWV. As described in Eq. (1), the stiffness of blood vessels is directly related to the PWV and vascular age in turn. The stiffness of blood vessels increases with aging, which leads to an increase in PWV^9,48^. The increase in PWV with aging applies equally to the clinically measured cfPWV and the estimated aPWV from the central aortic BP waveform.

### Change of blood pressure waveform according to cardiovascular parameters

Cardiovascular parameters include passive factors that depend on blood vessel, such as stiffness, diameter, and thickness; and, active factors that depend on cardiovascular conditions, such as HR, SV, and PR. The clinically observed trend of blood pressure change according to the cardiovascular parameters is shown in Fig. 2d. The waveform of central aortic BP according to age is shown on the left-hand side of Fig. 2d^5^. In the young-aged (19) BP waveform, two distinct peaks of forward and reflected waves are observed. In the middle-aged (42) BP waveform, which is the human standard BP waveform with SBP of 120 mmHg and BDP of 80 mmHg, AP is partially observed during systole due to the overlap of forward and reflected waves. In the old-aged (83) BP waveform, AP is observed in almost the entire area of systole due to the overlap of forward and reflected waves in wider region. As mentioned in the previous section, the stiffness of the vessel wall increases with aging, which leads to increase in PWV. With aging, the rupture and regeneration of elastic fibers of the inner layer are repeated, fibrosis and hardening proceed, and the composition ratio of the collagen fiber of the outer layer rises, resulting in increase of stiffness^5^. Clinically measured cfPWV values are 5–7 m/s for the young-aged, 6–9 m/s for the middle-aged, and 9–12 m/s for the old-aged^9,48–51^. The increased PWV with aging shortens the reflected wave's return time from AB, widens the overlapping region with the forward wave, and increases AP and SBP in turn. In other words, one of major causes of hypertension with aging is the increase in vascular stiffness and corresponding PWV.

The center of Fig. 2d shows that the two HR control methods (drug dosing and pacemaker) have different trends of SBP and DBP changes versus HR^14^. *Millasseau* studied the changes in BP by controlling HR using drugs and pacemakers separately. The reason for comparing the two HR control methods is that HR and SV cannot be controlled independently but changes simultaneously. Cardiac output (CO), i.e., the amount of blood pumped out per minute, is defined by the product of HR and SV. The CO is related to the amount of oxygen required depending on psychological state, exercise, and hormones, etc. When an adrenaline-like drug is dosed, the amount of required oxygen in the body is increased to have a large CO, i.e., HR and SV increase simultaneously. In this case, it is observed clinically that the SBP increases, and the DBP is maintained. On the other hand, when the HR is artificially controlled using a pacemaker, the amount of oxygen required in the human body does not change, so the CO remains constant, thus the SV decreases as the HR increases. In this case, it is observed clinically that the SBP is maintained and the DBP increased, which shows a different behavior compared to the case of drug dosing. This contradictory tendencies of BP change are evidence that multiple parameters influence human BP in a complex way. Both HR control methods increased MAP but showed opposite changes in PP.

2 $$MAP=\left(SBP+2\times DBP\right)/3$$

MAP is an area average for one period of the BP waveform, and is defined as Eq. (2) in a simplified expression. PP is the amplitude of the BP waveform and is the difference between SBP and DBP. Since the SV of the two HR control methods changes oppositely, it is estimated that MAP and PP are correlated with HR and SV, respectively, but it is impossible to evaluate the influence of individual parameters clinically.

PR means the hydrodynamic resistance acting in the circulation of blood in the cardiovascular system. The total peripheral resistance (TPR) is the sum of the PR of each blood vessel in the entire cardiovascular system, and it is defined as the ratio of MAP to CO. The TPR is an indicator of the pressure required to circulate blood with rate valued in CO [$\mathrm{mL}/\mathrm{min}$], the blood flow per minute. TPR increases as the cross-sectional area of the blood vessel decreases. In this study, PR is defined as the PR of the capillary parts of the TPR, excluding the aorta and arteries. On the other hand, Aortic resistance (AR) is defined as the PR of the aorta and arteries. TPR is the sum of AR and PR. A vein has a PR of 0 because the pressure difference is negligible. The right-hand side of Fig. 2d shows the change in BP according to the PR, reported in the study of Saugel and Schultz^56,57^. To control the PR, in case of injection level of the vasodilator increases, the vessel diameter increases, and the PR decreases. As PR decreases, a decrease in MAP and an increase in PP are observed. This is estimated to be caused by a decrease in TPR and a decrease in the ratio of PR among TPR, but there are limitations in clinical studies because other cardiovascular variables, such as HR, were not considered.

## Methods

### Overall design of cardiovascular simulator

**Figure 3:**
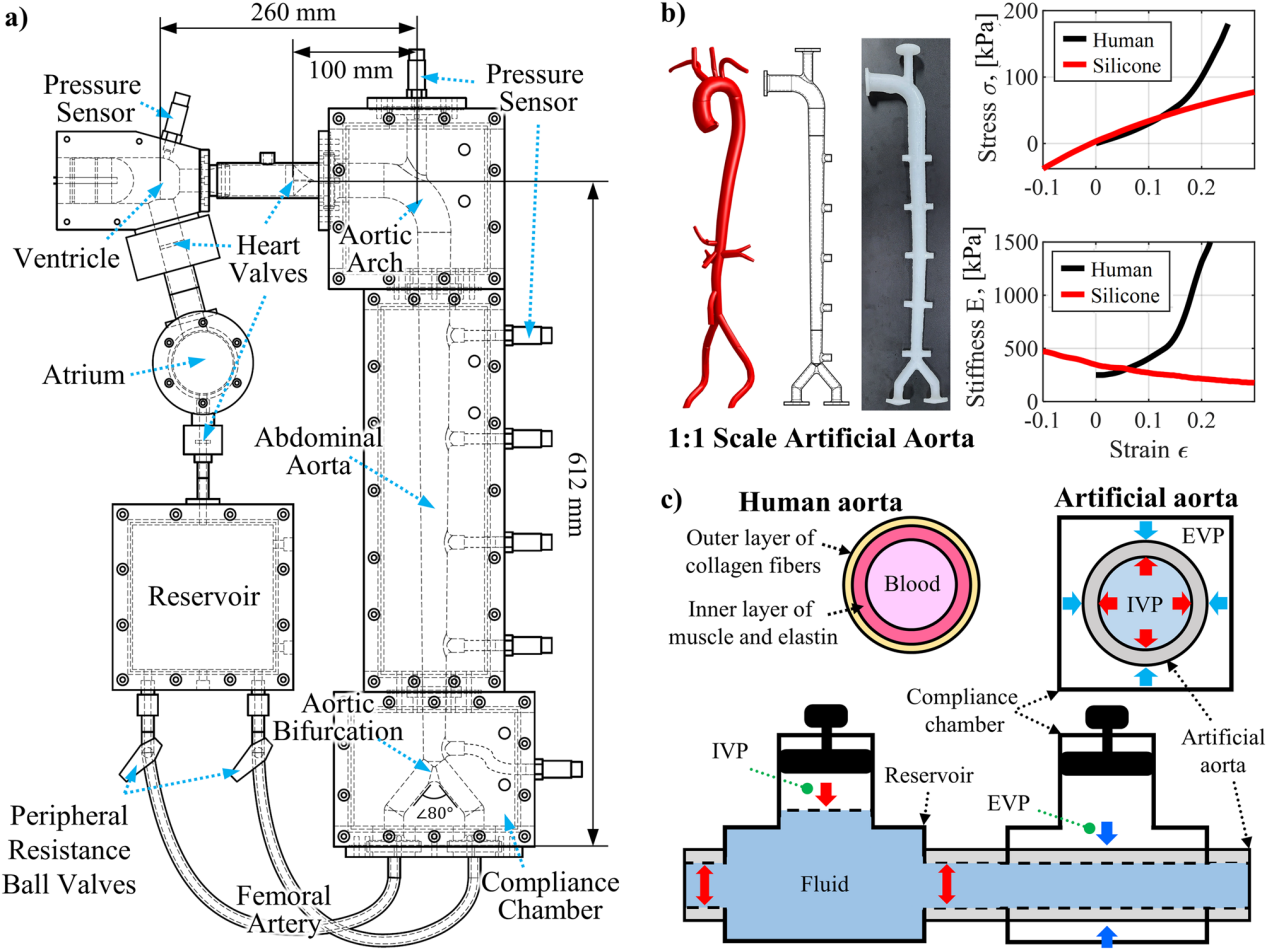
Layout of cardiovascular simulator; (**a**) detailed structure layout and name of each part of simulator, (**b**) fabrication of artificial aorta and its stiffness test result compare to human blood vessel, (**c**) schematic diagram of schematic diagram of the cross-sectional structures, the human and the artificial aorta, and the connection of the artificial aorta to the reservoir and compliance chamber.

Figure 3a shows the layout of cardiovascular simulator in this study mimicking the physiological structure and properties of the human cardiovascular system to reproduce the blood circulation and the propagation of BP waves inside the aorta. The forward wave is generated from a linear ventricular pump that acts as the left ventricle. The aortic valve is located next to the ventricular pump to prevent backflow. The aortic valve is made of soft silicone similar to the human’s manufactured in the form of a plate with a Y-shaped hole. After the aortic valve, an artificial aorta is connected. The artificial aorta mimics the shape and properties of the human AA, abdominal aorta, and AB. The entire artificial aorta is sealed in the compliance chamber, and connected to reservoir. The artificial aorta is pressurized from the inside and outside through the reservoir and compliance chamber, respectively, with a connected pneumatic pump. The detailed design of the artificial aorta is described in following section. After the artificial aorta, it is connected to two PR ball valves that control PR, then connected to a reservoir that acts as a simplified vein. The density of human blood ranges from 1043 to 1060 kg/m^3^, and the viscosity is around 3–4 cP^5^. In large-diameter aortas, the effect of inertia is significant, while the impact of viscosity is negligible^46,47^. Therefore, in this study, distilled water was used as the fluid inside the simulator since it has a 1000 kg/m^3^ density similar to blood and a 1 cP of viscosity has the same order of magnitude as that of blood.

During systole, the ventricular pump contracts, generating flow and forward pressure waves. The forward pressure wave propagates through the artificial aorta, overlaps with the reflected wave returning from the AB, and generates a BP waveform. Blood flow through the AB reaches the reservoir. During diastole, the ventricular pump expands, the aortic valve prevents backflow, and fluid moves from the reservoir through the atrium and into the ventricle. The cardiovascular simulator in this study controls HR and SV by changing the input function to the pump and controls PR by changing the PR ball valves occlusion ratios. In addition, the internal and external pressures of the artificial aorta are controlled using the reservoir and the compliance chamber with a connected pneumatic pump. To observe the blood pressure waveform according to the cardiovascular parameters, the pressure is measured with pressure sensors at ventricle and multiple points in the artificial aorta, compliance chamber, and reservoir. NI-9234 DAQ (National Instruments, USA) was used to acquire the pressure sensor data, the sampling rate was 8 kHz, and a 30 Hz low-pass filter was applied.

### Artificial aorta

The cardiovascular simulator includes the artificial aorta that mimics the physiological structure and physical properties of the human aorta. The artificial aorta was designed based on the 3D scanned shape and dimensions of the actual human central aorta with 1:1 scale, and some of peripheral structures, such as the renal artery and the right subclavian artery, were simplified^5^. The shape of the artificial aorta is shown on the left-hand side of Fig. 3b. The artificial aorta has total length of 743 mm, composed of the AA with radius of curvature of 50 mm, the abdominal aorta with length of 512 mm, and AB with angle of 80°. The inner diameter of the AA is 28 mm, the abdominal aorta is tapered from 26 to 16 mm in diameter, and the inner diameter of the femoral artery after the AB is 14 mm. The wall thickness of the entire vessel is 2 mm. The artificial aorta was manufactured by injecting silicone into a 3D printed mold, and PST 164 pressure sensors from EFE (France) for pressure measurement were installed in each part of the artificial aorta evenly distributed with interval of 100 mm. As mentioned before, the stiffness of the artificial aorta is important in reproducing the BP waveform, because it determines the PWV and the arrival time of the reflected wave. From the cfPWV, length and diameter of the vascular network, the aPWV, which determines the central aortic BP waveform, can be estimated. The calculated aPWV is about 87% of the cfPWV, because the femoral artery section with a small diameter and high PWV is excluded from the propagation path^58^.

In this study, the human standard BP waveform was mimicked as shown in Fig. 1b. An R-PTT of 0.2 s is required to reproduce this, and the target aPWV is calculated as 6.54 m/s. From Eq. (1), the stiffness of the silicone required to achieve the target aPWV value of 6.54 m/s is calculated as 350 kPa. The diameter and stiffness used for calculating PWV with Eq. (1) and manufacturing the artificial aorta in this study are clinically measured values of the in-vivo state^5,9,48–51^. Also, since these physical quantities change dynamically due to the IVP pulsating between the SBP and DBP, this study used pulse-averaged physical quantities which are averaged values on time within a single pulse. These values have already considered the effects of interactions with surrounding tissues and IVP-induced changes. The transmural pressure (TP), which is the difference between IVP and EVP, is similar to DBP and acts as an IVP at 80 mmHg based on negligible EVP in the body. TP determines the initial diameter and stiffness with expansion and strain-hardening. However, in this study, since the artificial aorta was already manufactured based on the modified diameter and stiffness due to IVP, pulse-averaged TP needs to be maintained at zero. To reproduce the human standard PWV and BP waveform, the artificial aorta was fabricated using SortaClear 12 biomimetic silicone manufactured by Smooth-On, Inc. (USA). To measure the stiffness of silicone used in the artificial aorta, a dog-bone specimen for tensile testing and a cylinder specimen for compression testing were fabricated simultaneously with the artificial aorta. The tensile and compression tests were performed using a push–pull gauge (DS2-500N, OPTECH, China). The tensile and compression test results of SortaClear 12 silicone used to fabricate the artificial aorta are shown on the right-hand side of Fig. 3b. The upper part is stress–strain curve, and the lower part is stiffness-strain curve measured using the slope of the stress–strain curve. The measured stiffness of SortaClear 12 is 346 kPa at zero strain, which is similar to the target stiffness of 350 kPa^59,60^.

However, biomimetic silicones, including SortaClear 12, have some different physical properties and behavior from those of human blood vessels. Human blood vessels have a complex structure with inner wall of elastin and muscle cells and an outer wall of collagen, as shown on the left-hand side of Fig. 3c. Therefore, as shown in the black line in the right-hand side of Fig. 3b, it shows a strain-hardening characteristic in which the stiffness increases as it stretches^59^. This is one of the reasons why blood vessels do not rupture even when high blood pressure is present in the body. As blood pressure increases, the stiffness of blood vessels increases rapidly due to strain-hardening properties, and the diameter of blood vessels does not increase as much as the stiffness, leading to the observation of rapid PWV^45^. On the other hand, in the case of biomimetic silicone, as shown on the red line in the right-hand side of Fig. 3b, it has a strain-softening characteristic in which the stiffness decreases as the strain increases. This is a characteristic found in most biomimetic silicones as well as SortaClear 12^61,62^. Thus, silicone has a similar stiffness to human blood vessels up to 10% tensile and compressive strain, but expands as the IVP increases, and there is a risk of over-expansion and rupture due to excessive strain-softening effect. The stiffness softening of silicone must be suppressed to reproduce PWV and BP waveforms similar to human. In this study, a specially designed compliance chamber was introduced to maintain the TP of the artificial aorta at zero and prevent strain-softening effects.

### Compliance chamber

The compliance chamber in this study was designed to control the EVP of the artificial aorta. It is used to prevent strain-softening, along with a reservoir that controls the IVP of the artificial aorta. The strain-softening of silicone changes the stiffness and makes it impossible to reproduce the desired PWV and even BP waveforms similar to human. When reproducing the BP waveform in the simulator, the IVP of the artificial aorta is raised using a reservoir to move the pressure to the same as the DBP of a human. In the case of the human blood vessel, this increase in IVP would lead to an increase in diameter and a rapid increase in stiffness due to strain-hardening, resulting in an increase in PWV. However, in the artificial aorta, the biomimetic silicone with strain-softening behavior causes a rapid increase in diameter and a decrease in stiffness, which leads to a rapid decrease in PWV. This distorts the BP waveform, and severe stiffness decreases can lead to rupture of the artificial aorta. To mimic the strain-hardening of human blood vessel walls, composite layers in which silicone or fiber meshes with different stiffnesses are multi-layered have been proposed^39,41^. However, applying the composite layers to mimic the complex blood vessel structures is one of challenging tasks due to many of design variables in nonlinear relations.

In this study, instead of developing the multi-layered tube, the outside of the artificial aorta is pressurized with air by the compliance chamber to compensate the strain-softening effect of the biomimetic silicone. The compliance chamber uses air, which has a negligible density compared to water, for EVP control. This minimizes contact, interaction, or loading with the artificial aorta because the changes caused by IVP and interactions with surrounding tissues have already been considered in manufacturing the artificial aorta. The compliance chamber seals the entire exterior of the artificial aorta, and the working principle is on the right-hand side of Fig. 3c. The compliance chamber applies the same level of EVP to the artificial aorta as the IVP applied by the reservoir. As a result, pulse-averaged TP becomes zero, expansion of the artificial aorta is suppressed, strain is maintained at 0, and strain-softening is prevented. The compliance chamber maintains the desired stiffness of the artificial aorta, which enables the reproduction of PWV and BP waveforms close to human. Also, as long as IVP and EVP are kept the same, we can simply adjust the level of the BP waveform in a controllable manner close to human cardiovascular system.

## Results

### Reproduction of blood pressure waveform

**Figure 4:**
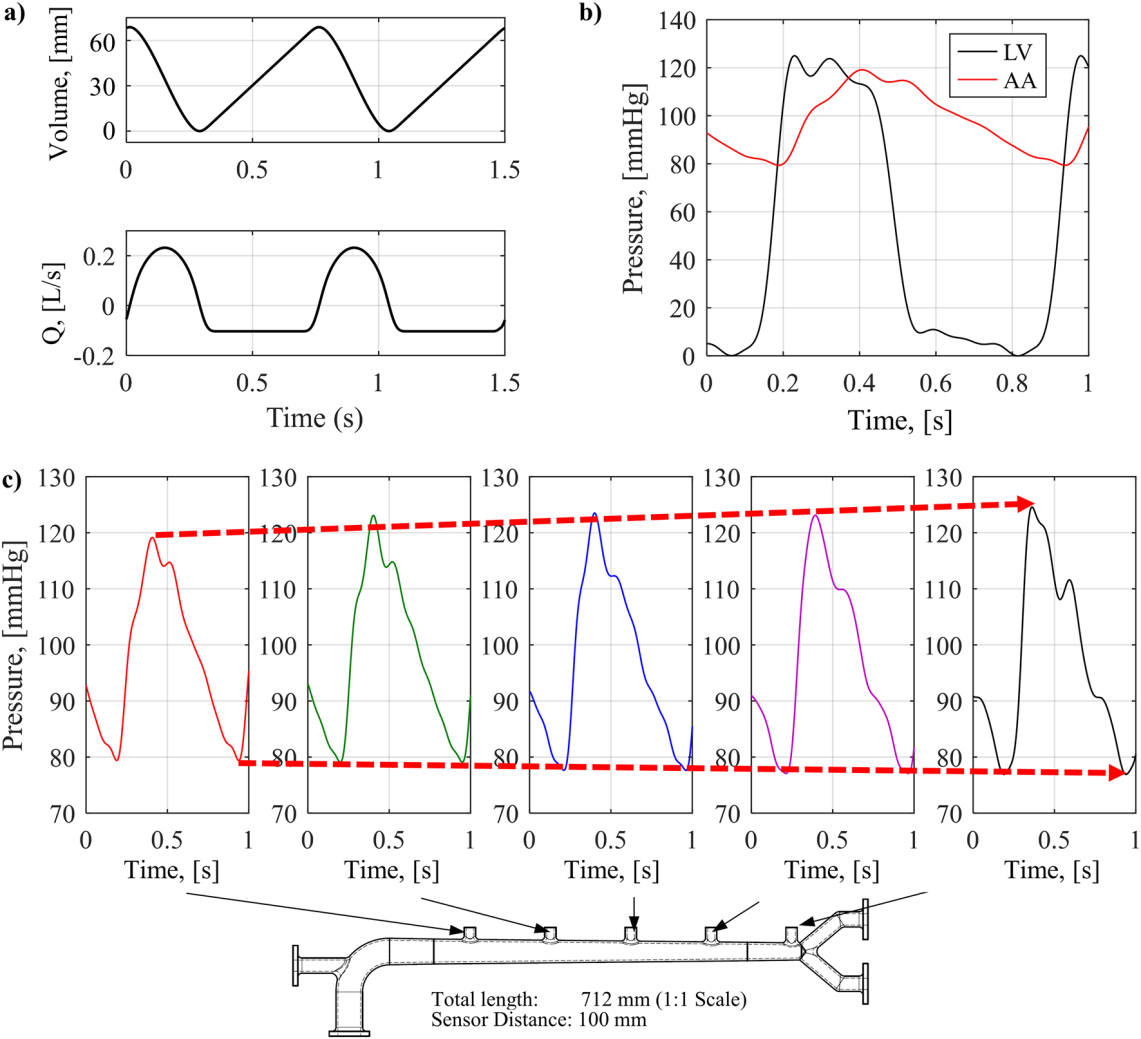
The reproduced BP waveforms by the cardiovascular simulator; (**a**) the volume-time curve and flow rate profile of the ventricular pump, (**b**) pressure waveforms in the ventricle and aortic root, (**c**) Change of BP waveform according to the measurement position in the artificial aorta.

Figure 4 shows the BP waveform reproduced by the cardiovascular simulator. Figure 4a shows the volume-time curve applied to the ventricular pump. The volume-time curve is an integral form of the blood flow rate of a human. This volume-time curve is proper to reproduce magnitudes and waveforms of ventricular and aortic pressures. The pulse frequency is 75 BPM, and the volume ejected per pulse is 70 mL, which is the same as human standard HR and SV^5^. Figure 4b shows the pressure waveforms in the ventricle and AA. The ventricular pressure waveform has a maximum value of 120 mmHg and a minimum value of 0 mmHg. The central aortic BP waveform measured in the artificial aorta has SBP 120 mmHg and DBP 80 mmHg, with less than 1 mmHg error from the human standard. This error is smaller than the tolerance of 5 mmHg for ESH protocol A-grade blood pressure monitors^63^. The MAP of the reproduced central aortic BP waveform is also 93.3 mmHg same as the human standard^5^.

As shown in Fig. 4c, as the BP waveform propagates and approaches the AB, the SBP increases, the DBP decreases, and the PP increases. Also, the region where the augmentation is formed due to the overlap of the reflected wave in the systolic peak clearly increases. This is the same as the change of BP waveform according to propagation in the human aorta, shown in Fig. 2b. Using the time interval of the BP waveforms between the aortic root and the AB from the pressure sensor mounted on the artificial aorta, aPWV can be measured. The measured aPWV of the cardiovascular simulator was 6.58 m/s, which is very close to the target aPWV value of 6.54 m/s with an error of 0.05 m/s. This results in a PTT error of less than 1 ms for an aortic length of 712 mm.

Therefore, the cardiovascular simulator in this study successfully reproduced the SBP, DBP, and PWV of the human standard. PP was 40 mmHg, and AP, the pressure difference between the inflection point and the peak, was 5.3 mmHg, so AIx was calculated as 13.3%. This value is in the middle of the human standard AIx range (11–16%)^42^. Precise PWV and BP level reproduction enables reproduction of BP waveforms similar to humans. In the following section, based on the BP waveforms with 120 mmHg SBP and 80 mmHg DBP reproduced by the cardiovascular simulator, changes of the BP waveform according to cardiovascular parameters are investigated. By controlling cardiovascular parameters such as HR, SV, PR, IVP, and EVP, the change in BP waveform is observed and compared with human characteristics under the similar changes of cardiovascular parameters.

### Blood pressure waveform versus heart rate and stroke volume

**Figure 5:**
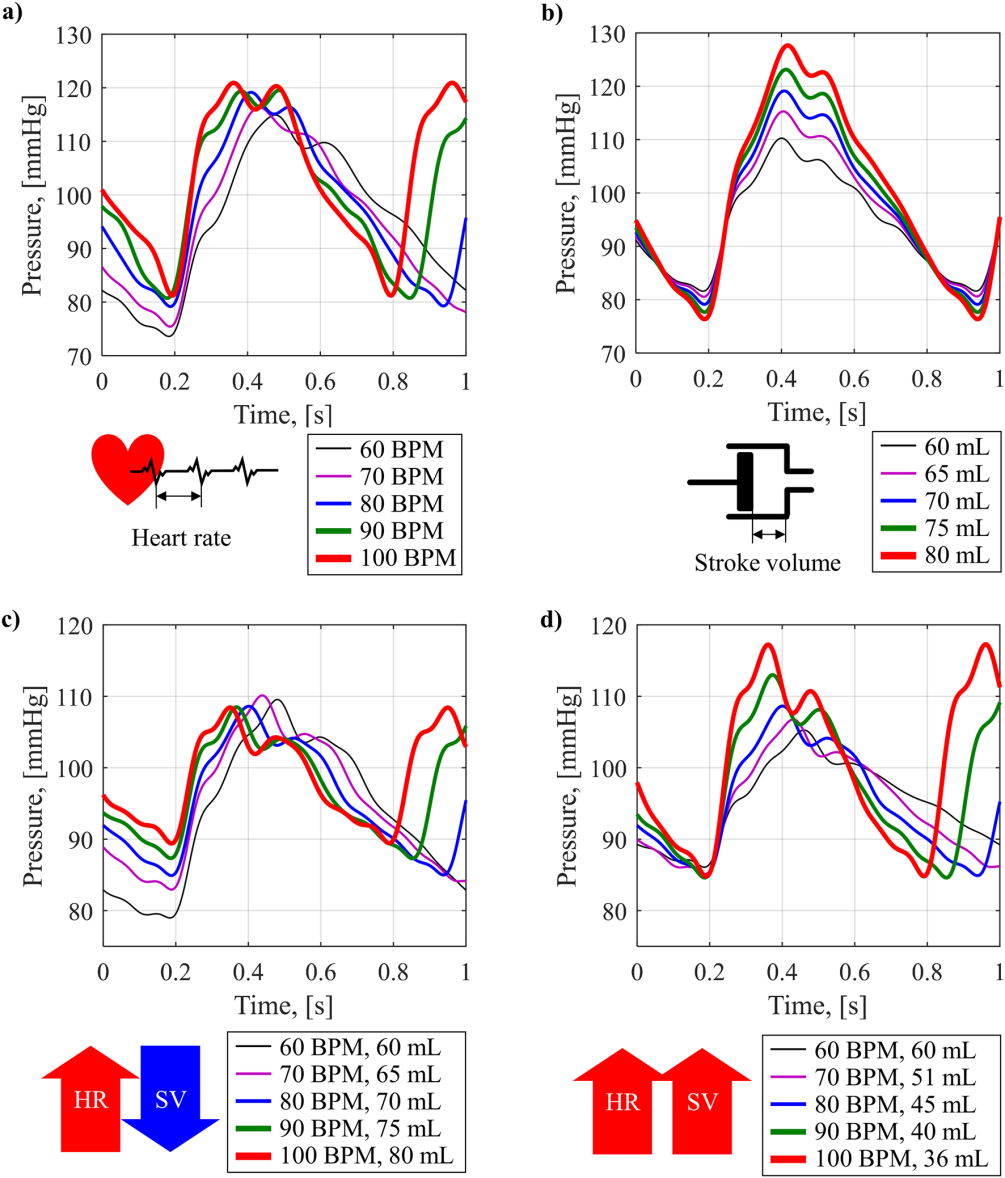
Change in BP waveform with HR and SV; (**a**) change in BP waveform with increasing HR, (**b**) change in BP waveform with increasing SV, (**c**) change in BP waveform when increasing HR and decreasing SV while maintaining CO, (**d**) change in BP waveform when increasing HR and SV simultaneously, while increasing CO.

Figure 5 shows the BP waveforms reproduced by the cardiovascular simulator while controlling HR and SV. HR and SV are controlled through the ventricular pump drive profile in the cardiovascular simulator. The pulsation period of the pump piston determines the HR, and the stroke length determines the SV. In Fig. 5a, the change in BP waveform was observed while the HR was increased from 60 to 100 BPM by 10 BPM. As HR increased, SBP and DBP increased, resulting in increased MAP. In Fig. 5b, the change in BP waveform was observed while the SV was increased from 60 to 80 mL by 5 mL. As SV increased, SBP increased significantly, and DBP decreased, resulting in maintained MAP and increased PP.

By simultaneous control of HR and SV, the change of the BP waveform was observed. Figure 5c shows the change of BP waveform when HR increases and SV decreases while maintaining a constant CO. CO is held at 3.6 L/min, HR is increased from 60 to 100 BPM in 10 BPM increments, and SV is decreased to maintain CO. As HR increased while maintaining CO, SBP was maintained, but DBP increased, resulting in an increase in MAP and a decrease in PP. Figure 5d shows the change of BP waveform when HR and SV increase simultaneously, in turn, CO increasing. HR is increased from 60 to 100 BPM by 10 BPM, and SV is increased from 60 to 80 mL. As HR and SV increased so that CO increased, SBP was increased, and DBP was maintained, so MAP and PP increased. These two experimental cases showed the same behaviors to human BP waveforms according to the changes of CO and HR in cases of pacemaker and drug dose, which are described in theoretical background (the center of Fig. 2d). As result, the change in SBP and DBP of a human when CO changes according to HR and SV by pacemakers and drugs were reproduced similarly by the cardiovascular simulator. In addition, changes in the BP waveform according to changes in individual parameters of HR and SV, which is very difficult to be performed clinically, can be observed in a controllable way. From the experiment, it was demonstrated that HR determines MAP; and SV determines PP; and also that the two cardiovascular parameters have complicated effects on BP waveform generation.

### Blood pressure waveform versus peripheral resistance

**Figure 6:**
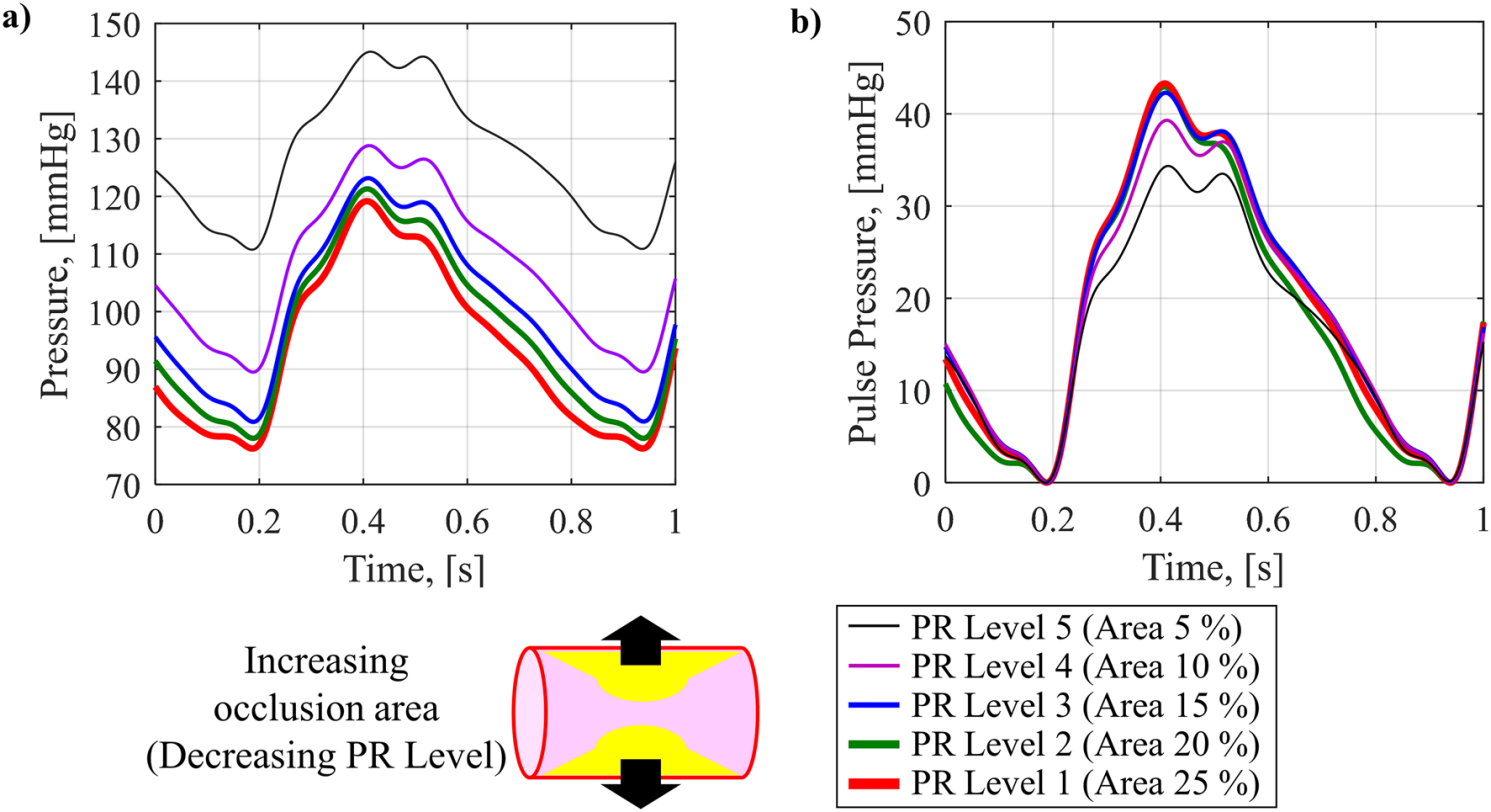
Change in BP waveform with PR; (**a**) BP waveforms with decreasing PR, (**b**) change in PP with decreasing PR.

Figure 6 shows the change of the BP waveform reproduced by the cardiovascular simulator while controlling PR. PR is controlled by the PR ball valves mounted on the reservoir in charge of veins in the cardiovascular simulator. Changing the occlusion ratio of the femoral artery leading from the AB using a PR ball valves make it possible to control the sectional area from full opening to complete closing. To reproduce the standard BP waveform, the appropriate value for the occlusion ratio of the PR ball valve in the simulator was 15%. Using this ratio as a baseline, the ball valve was controlled to change the occlusion rate by 5% from 5 to 25% to observe the change in the BP waveform according to the PR decrease over 5 stages. In Fig. 6a, the change in BP waveform was observed when PR was decreased by controlling the occlusion ratio. As PR decreased, SBP and DBP decreased sensitively, decreasing MAP. Figure 6b shows only PP by subtracting DBP from each BP waveform. As PR decreases, PP increases. In summary, when PR decreases, MAP decreases and PP increases in the BP waveform reproduced by the cardiovascular simulator. From the trend of MAP decrease and PP increase according to PR decrease using the vasodilator shown in theoretical background (the right-hand side of Fig. 2d).

### Blood pressure waveform versus intravascular and extravascular pressures

**Figure 7:**
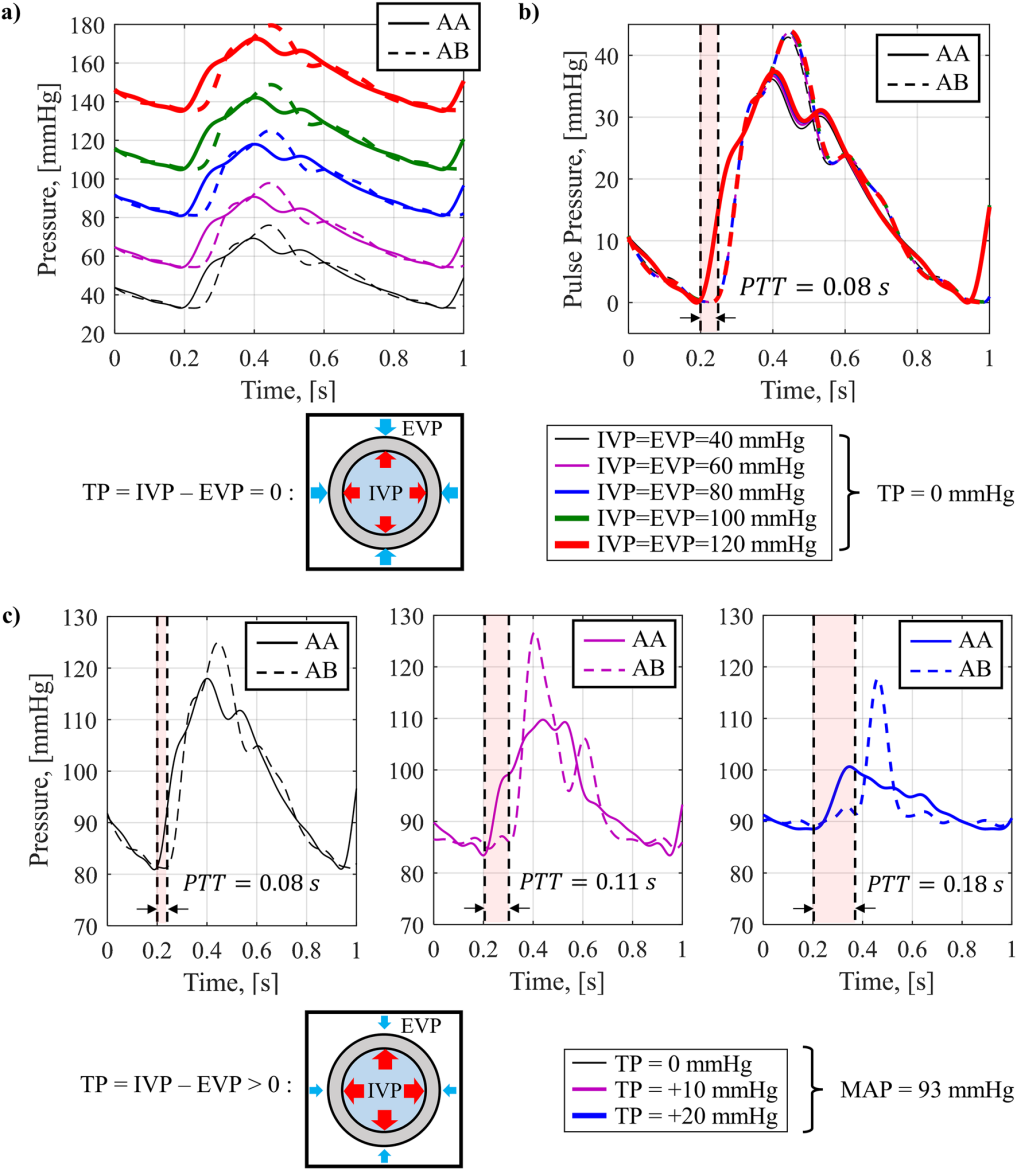
Change in BP waveform with IVP and EVP; (**a**) change in wave at aortic arch (AA) and aortic bifurcation (AB) with increasing IVP and EVP, (**b**) change in PP of AA and AB with increasing IVP and EVP, (**c**) change in wave at AA and AB with increasing TP.

The cardiovascular simulator in this study includes the dedicated compliance chamber. In the artificial aorta, the reservoir increases BP to the same level as humans by applying IVP, and the compliance chamber prevents strain-softening by applying EVP to suppress expansion by air pressure. Using a cardiovascular simulator, through the control of IVP and EVP, the change in BP waveform according to TP was observed. Figure 7 shows the change in BP waveform in AA and AB while controlling TP. Figure 7a shows the waveforms when TP is 0, which is IVP and EVP are equally controlled from 40 to 120 mmHg at intervals of 20 mmHg. Figure 7b shows corresponding PP by subtracting each BP waveform in Fig. 7a by DBP. When TP is 0, the BP waveform reproduced by the simulator only shifts the pressure level without changes in the waveform and PP. The PTT, the difference in arrival time of the BP waveforms measured in AA and AB, was calculated based on the lowest point of each waveform and was maintained even when IVP increased. This implies that when the compliance chamber maintains TP at 0, even if IVP is changed, PWV and vascular stiffness remain unchanged from the target value.

However, if TP is greater than zero, the IVP generated by the reservoir is greater than the EVP generated by the compliance chamber. Figure 7c shows the change in the BP waveform when the TP is increased while maintaining the MAP of 93 mmHg, the same as that of a human. TP was increased from 0 to 20 mmHg in 10 mmHg steps. The experiment was stopped for TP values higher than 20 mmHg due to concerns about the potential rupture of the artificial aorta. Figure 7d shows the PP by subtracting each BP waveform by DBP. As TP increased, SBP decreased sensitively, and DBP increased. Therefore, the PP decreased, and the waveform of the BP waveform also drastically changed. It is also observed that the arrival time difference between AA and AB, PTT, increases with TP. Table 1 summarizes the average values of PTT, PWV, and stiffness E estimated through Eq. (1) for 5 pulses as TP increases. As TP increases, PTT increases, PWV decreases, and it is confirmed that the estimated stiffness rapidly decreases. This is because the EVP generated by the compliance chamber is insufficient to prevent artificial aorta expansion and strain-softening caused by the IVP generated by the reservoir. The stiffness of the artificial aorta decreased due to strain-softening, and the PWV decreased due to expansion and weakened stiffness, which is the root causes of the change in the BP waveform and the decrease in PP, increase in PTT.

**Table 1:** PTT, PWV and stiffness decrease due to strain-softening with increasing TP (average of 5 times).

TP [mmHg]	Dist. [mm]	PTT [s]	PWV [m/s]	Est. E [kPa]
0	551	0.0838	6.58	346
10	551	0.1079	5.11	248
20	551	0.1763	3.13	80

## Discussion

The presented cardiovascular simulator can reproduce a human BP waveform with a SBP of 120 mmHg and a DBP of 80 mmHg under the condition of 75 BPM HR and 70 mL SV pulsation. The measured PWV of 6.58 m/s, and AIx of 13.3%, which is similar to the clinical value of middle-aged^5,9,48–51^. These values are in the middle of the human standard range, and the reproduced blood pressure waveform is similar to that of humans. The errors from the human standard values are less than 1 mmHg for BP, less than 0.05 m/s for PWV, and less than 3% for AIx. The simulator reproduced not only the standard BP waveform but also the BP waveform changes according to cardiovascular parameters such as HR, SV, and PR observed in humans. In contrast to the human body, where HR and SV cannot be independently controlled, the simulator in this study allows for independent or simultaneous control of HR and SV. HR was adjusted at 10 BPM intervals from 60 to 100 BPM, and SV was adjusted at 5 mL intervals from 60 to 80 mL. Individual parameter control experiments showed that HR was involved in MAP, and SV was involved in PP. With simultaneous control of HR and SV, the simulator reproduced the changes in BP under conditions, which are the increase in CO due to drug administration and the maintenance of CO due to pacemaker stimulation^14^. In addition, by controlling the valve occlusion area at the end of the femoral artery, the simulator reproduced the changes in BP waveform according to PR. The occlusion area of the PR valve was increased from 5 to 25% in 5% increments. A decrease in PR resulted in a decrease in MAP and PP, which is similar to the change observed in humans when PR is decreased by administering vasodilators that increase the cross-sectional area of capillaries^56,57^. The simulator uses a compliance chamber to apply the same EVP as the IVP, which sets TP to 0 and suppresses the expansion of the artificial aorta to prevent changes in stiffness and PWV and distortion of the BP waveform due to strain-softening. As a result of the experiment, with zero TP condition, it was observed that a constant PWV and a BP waveform without distortion were maintained even when the DBP level was controlled from 40 to 120 mmHg. On the other hand, with positive TP condition up to 20 mmHg, the EVP given by the compliance chamber is insufficient, and the artificial aorta expands, strain-softening occurs, resulting in a decrease in stiffness and PWV and distortion of the BP waveform.

In this study, the simulator features an artificial aorta with a 1:1 scale shape and similar vascular stiffness to reproduce PWV and generate the BP waveform. Previous studies on simulators for blood pressure waveform reproduction have used different methods for generating blood pressure. Some simulators only implement the BP ventricle and atrium without the aorta and adjust the input volume-time curve^15,16,25–28^. Some others implemented an aorta structure but used materials like acrylic, PVC, or soft silicone but not in the physiological range^18,32–36^. However, these methods cannot reproduce PWV, the critical physical property involved in generating blood pressure in humans. There have been studies attempting to manufacture artificial blood vessels that have similar physical properties to those of humans. However, due to the complex manufacturing process that involves mixing or multi-layering various materials, the resulting artificial vessels are limited in shape to either a plate or a cylindrical shape^37–41^. It is difficult to replicate the size and structure of a human aorta using these artificial vessels. In contrast, the simulator in this study implemented an artificial aorta that mimics the shape and stiffness of a human, and it is possible to reproduce a BP waveform with the same magnitude and shape as a human by maintaining the stiffness through a compliance chamber. The simulator is able to reproduce not only the magnitude and shape of the BP waveform but also the changes observed with variations in cardiovascular parameters because it reproduced the process of BP waveform generation through the superposition of forward and reflected waves. The simulator demonstrated and investigated with physically explainable mechanisms, the changes in BP waves according to the controllable cardiovascular parameters that are difficult to control in clinical studies. Expanding the design of the simulator can lead to more clinical and practical applications. Currently, the artificial aorta of the simulator is manufactured with middle-aged stiffness to reproduce the standard central BP waveform, but artificial aortas with various stiffnesses are required to study the change of BP waveform according to aging. Diversification of the artificial aorta according to stiffness will be the basis for reproducing aging and vascular diseases such as aortic aneurysm and arteriosclerosis and their symptoms. In addition, expanding the simulator to other arteries can enable the measurement of blood pressure in the brachial artery and the generation of PPG in the radial artery. Also, in contrast to the aorta, PWV and BP waveforms in small-diameter arteries are influenced by viscosity, which can be useful in studying conditions related to blood health, such as hyperlipidemia^7,44–47^. This would enable the simulator to be a preclinical testing platform for developing smart wearable devices or medical instruments.

## Conclusion

In this paper, the presented cardiovascular simulator reproduces the human BP waveform and behaviors according to cardiovascular parameters in same pressure range and trends. The cardiovascular simulator in this study is applicable to supplement missing links in clinical data to collect a massive dataset and analyze the mechanism of cardiovascular diseases and symptoms. However, the artificial aorta of the simulator has a limitation in that it cannot reproduce the BP waveform of the brachial or radial artery, which is frequently measured and applied. In the future, by replacing the artificial aorta with properties of young and old-aged, it can be expanded to study BP waves according to age. In addition, it can be applied to developing BP measuring instruments and wearable devices, e.g. cuffs and smartwatches by extending the vascular system of the brachial artery and radial artery from the artificial aorta in the future works^64,65^.

## Data Availability

The datasets generated during and/or analyzed during the current study are available from the corresponding author upon reasonable request.

## Abbreviations

BP: Blood pressure
SBP: Systolic blood pressure
DBP: Diastolic blood pressure
MAP: Mean arterial pressure
PP: Pulse pressure
PWV: Pulse wave velocity
PTT: Pulse transit time
R-PTT: Reflected PTT
AP: Augmented pressure
AIx: Augmentation index
HR: Heart rate
SV: Stroke volume
CO: Cardiac output
TPR: Total peripheral resistance
AR: Aortic resistance
PR: Peripheral resistance
IVP: Intravascular pressure
EVP: Extravascular pressure
TP: Transmural pressure
cfPWV: Carotid-femoral PWV
aPWV: Aortic PWV
LV: Left ventricle
AA: Aortic arch (ascending aorta)
AB: Aortic bifurcation
